# Comparison of Different Scoring Systems Based on Both Donor and Recipient Characteristics for Predicting Outcome after Living Donor Liver Transplantation

**DOI:** 10.1371/journal.pone.0136604

**Published:** 2015-09-17

**Authors:** Yucheng Ma, Qing Wang, Jiayin Yang, Lunan Yan

**Affiliations:** Department of Liver Transplantation, West China Hospital of Sichuan University, Chengdu, China; University of Toledo, UNITED STATES

## Abstract

**Background and Objectives:**

In order to provide a good match between donor and recipient in liver transplantation, four scoring systems [the product of donor age and Model for End-stage Liver Disease score (D-MELD), the score to predict survival outcomes following liver transplantation (SOFT), the balance of risk score (BAR), and the transplant risk index (TRI)] based on both donor and recipient parameters were designed. This study was conducted to evaluate the performance of the four scores in living donor liver transplantation (LDLT) and compare them with the MELD score.

**Patients and Methods:**

The clinical data of 249 adult patients undergoing LDLT in our center were retrospectively evaluated. The area under the receiver operating characteristic curves (AUCs) of each score were calculated and compared at 1-, 3-, 6-month and 1-year after LDLT.

**Results:**

The BAR at 1-, 3-, 6-month and 1-year after LDLT and the D-MELD and TRI at 1-, 3- and 6-month after LDLT showed acceptable performances in the prediction of survival (AUC>0.6), while the SOFT showed poor discrimination at 6-month after LDLT (AUC = 0.569). In addition, the D-MELD and BAR displayed positive correlations with the length of ICU stay (D-MELD, p = 0.025; BAR, p = 0.022). The SOFT was correlated with the time of mechanical ventilation (p = 0.022).

**Conclusion:**

The D-MELD, BAR and TRI provided acceptable performance in predicting survival after LDLT. However, even though these scoring systems were based on both donor and recipient parameters, only the BAR provided better performance than the MELD in predicting 1-year survival after LDLT.

## Introduction

Given the increasing demand for liver transplantation, deceased organs have not been able to meet the need of liver transplantation. Living donor liver transplantation (LDLT) provides a lifesaving alternative to medical therapy for patients with a variety of end-stage liver diseases. In order to optimize the outcome of LDLT, a scoring system that can accurately predict survival of patients undergoing LDLT is needed.

The Model for End-stage Liver Disease (MELD) based evaluation system was first formulated to predict the survival of patients with cirrhosis undergoing transjugular intra-hepatic portosystemic shunt in 2000 [1]. Since 2002, the MELD score has been implemented in the United States for predicting the survival after liver transplantation and in most western countries thereafter. This score is based on three widely available recipient variables, including the serum bilirubin, serum creatinine and international normalized ratio (INR).

In recent years, some new scoring systems based on both donor and recipient characteristics have been proposed to improve the discriminatory ability of survival prediction after liver transplantation. In 2008, Rana et al. developed the score to predict Survival Outcomes Following Liver Transplantation (SOFT) that contains both donor and recipient information to evaluate transplants at the time of transplantation [2]. D-MELD, the product of preoperative MELD and donor age was developed by Halldorson et al. to provide a better prediction of post-operative mortality and length of stay in 2009 [3]. With the desire for a balance between need and utility in organ allocation, the Balance of Risk (BAR) score has been developed by Dutkowski et al. in 2011 [4]. Recently, the Transplant Risk Index (TRI) was proposed, of which identified variables not captured in United Network for Organ Sharing (UNOS) that significantly correlated to graft failure [5].

The goal of this article was to evaluate and compare the performance of the four scoring systems, including D-MELD, SOFT, BAR, and TRI as predictive models for post-transplant outcome in patients undergoing LDLT.

## Patients and Methods

### Study Population

Between January 2001 and May 2014, 329 consecutive patients with end-staged liver diseases received LDLT in the West China Hospital of Sichuan University. The initial 15 LDLT transplants at our center were excluded, because of the recognized learning curve and change in post-transplant outcomes with increasing center experience [6,7]. Patients with malignant liver disease were included, however, MELD score was computed without inclusion of exception points awarded for a cancer diagnosis. After excluding pediatric recipients (<18 years of age, n = 62) and recipients with missing calculated MELD score (n = 3), a total of 249 adult patients were retrospectively analyzed. The outcome was assessed referred to 1-, 3-, 6-month and 1-year mortality rate. All of the data were obtained from the China Liver Transplant Registry System (CLTRS): http://www.cltr.org.

### Ethics Statement

This retrospective study was approved by the West China Hospital Ethical Committee, and it was carried out according to the principles expressed in the Declaration of Helsinki. The transplanted organs used in this study were obtained from close relatives of the recipients in West China Hospital of Sichuan University. The donors and their families were informed about the possible risks of donor hepatectomy before surgery. And the procedures of organ procurement were all conducted with the consent from donors. None of the transplant donors were from a vulnerable population and all donors or next of kin provided written informed consent that was freely given. All of the donors and recipients were anonymous in this study.

### Surgical Technique

The left or right portal vein and the left or right hepatic artery in donors were dissected with the help of intraoperative ultrasonography. And also, the hepatic vein was isolated. Intraoperative cholangiography was performed in donors before hepatectomy, in order to know the branches of hepatic biliary system. Aiming to find the inferior left or right hepatic vein, the secondary portal of liver was exposed by dividing the tissue between liver and diaphragm. Next, we sharply cut the left or right bile duct. After that, the donor hepatectomy was performed with a Cavitron Ultra-sonic Surgical Aspirator (CUSA System 200; Valleylab Inc., Boulder, CO) and an argon knife. Then, the graft was flushed with 4°C University of Wisconsin solution through two channels, the portal veins (PV) and the hepatic artery. Then the revascularization of the graft, arterial, biliary and portal anastomosis was performed. The graft outflow reconstruction has been carefully reported in our previous study [8]. Meanwhile, the recipient liver was resected. Following of which, using the piggy-back technique, the graft was orthotopically transplanted. End-to-end right portal vein anastomosis was used with continuous suture. Next, the hepatic artery anastomosis was conducted by interrupted suture. Last, either Roux-en-Y hepaticojejunostomy or duct-to-duct anastomosis was used to reconstruct the bile duct [9].

### Calculation of the MELD, D-MELD, SOFT, BAR, and TRI

The MELD score was computed according to the following equation: MELD = 0.957×loge[Creatinine (mg/dL)] + 0.378×loge [total Bilirubin (mg/dL)] + 1.12×loge (INR) + 0.643, where INR = international normalized ratio [10].

D-MELD is the product of donor age and preoperative MELD [3]. In the original article reported by Rana et al., the SOFT score took account of 18 risk factors from both donor and recipient, including the cause of donor’s death and whether allocated regionally or nationally [2]. However, for the reason that no deceased donors were included in this study, these two factors, cause of donor’s death and national allocation, were not taken into consideration in this analysis. We calculated the SOFT score by the rest 16 factors. The BAR score was calculated as described before [4]. The TRI was calculated by the formula: TRI = Exp [(0.008 × Donor Age) + (0.013 × Donor Peak Na) + (0.041 × Ischemia Time) + (0.070 × Recipient Creatinine) + (0.041×Recipient INR) + (- 0.006 if Recipient Hep C) + (- 0.021 × Donor Height × 0.393701)] where Hep C = positive for hepatitis C [5].

### Statistical Analysis

Continuous variables were expressed by mean values and standard deviations. And comparisons of the mean values were accomplished using a two-tailed Student’s test. Categorical data, reported as the number of cases and percentages, were compared with the Fisher’s exact test or the Pearson’s chi-square test as appropriate. To evaluate the capability of each score in predicting survival of LDLT patients, our study was performed by using receiver operating characteristic curves (ROC)[11]. The area under the ROC curve (AUC) was used to calculate the overall correctness of each score. The comparison of the AUCs from different scores and examination of the statistical significance of the AUCs were performed with the method of Hanley and McNeil [11]. The significance level was set at a p < 0.05. The data analysis was performed with the SPSS for Windows version 21 release (SPSS, Inc., Chicago, Illinois, USA) and MedCalc for Windows version 13.1.2.0 (MedCalc Software, Mariakerke, Belgium).

## Results

We list the patients’ characteristics in Table 1. Of the 249 patients, 216 (86.7%) were male and the mean age was 42.5 ± 8.9 years. And 194 (77.9%) patients had evidence of chronic hepatitis virus infection, including 186 (74.7%) HBsAg-positive subjects. Hepatitis C infection was found in 6 (2.4%) patients and dual hepatitis B and C infection was detected in another 2 (0.8%) patients. Among these patients, 126 (50.6%) had malignant tumors, including 105 (42.2%) patients with primary hepatocellular carcinoma (HCC), 14 (5.6%) patients with recurrent HCC after hepatectomy, 6 (2.4%) patients with cholangiocarcinoma, and one patient with both HCC and cholangiocarcinoma. Only 150 patients had the information of relationship between donors and recipients. We found that siblings was the biggest group to donate their livers (48/150, 32.0%), followed by nephews and nieces (21/150, 14.0%), cousins (20/150, 13.3%), spouses (20/150, 13.3%), children (19/150, 12.7%), parents (17/150, 11.3%), and uncles and aunts (5/150, 3.3%). Most of grafts were right lobe without middle hepatic vein (MHV), accounting for 93.2% (232), while 4.8% (12) were right lobe with MHV, 1.2% (3) were left lobe without MHV. Only two grafts were extended left lateral lobe (0.4%) and left lateral lobe (0.4%). Preoperative scores were: MELD, 15.8out middle hepatic vein (MHV), accounting for 93.2% (232), while 8.3. The median follow-up was 12.1 months (range = 0.1–99.7 months). The 1-, 3-, 6-month and 1-year overall survival rates were 90.8% (226/249), 87.1% (217/249), 84.7% (211/249), and 82.7% (206/249), respectively.

**Table 1:** Clinical features of the study patients.

Characteristics	Total patient population (n = 249)
Age (years)	42.5±8.9
Male gender	216 (86.7%)
Etiology of liver disease	
Virus	194 (77.9%)
HBV	186 (74.7%)
HCV	6 (2.4%)
HBV+HCV	2 (0.8%)
Alcohol	6 (2.4%)
Virus + alcohol	1 (0.4%)
Others	48 (19.3%)
Malignant	126 (50.6%)
Primary HCC	105 (42.2%)
Recurrent HCC after hepatectomy	14 (5.6%)
Cholangiocarcinoma	6 (2.4%)
Primary HCC and cholangiocarcinoma	1 (0.4%)
Donor's relation to recipient*	
Siblings	48/150 (32.0%)
Nephews and nieces	21/150 (14.0%)
Cousins	20/150 (13.3%)
Spouses	20/150 (13.3%)
Children	19/150 (12.7%)
Parents	17/150 (11.3%)
Uncles and aunts	5/150 (3.3%)
Type of graft	
Right lobe without MHV	232 (93.2%)
Right lobe with MHV	12 (4.8%)
Left lobe without MHV	3 (1.2%)
Extended left lateral lobe	1 (0.4%)
Left lateral lobe	1 (0.4%)
Time of hospitalization (days)	50.2±28.3
Length of ICU stay (days)	12.7±11.9
Time of mechanical ventilation (days)	0.8±1.3
Scores	
MELD	15.8±9.4
D-MELD	570.2±398.7
SOFT	1.6±3.7
BAR	3.9±4.2
TRI	5.4±8.3

NOTE: Values are expressed as number (%) or mean±SD. Abbreviations: SD, standard deviation; HBV, hepatitis B virus; HCV, hepatitis C virus; HCC, hepatocellular carcinoma; MHV, middle hepatic vein; ICU, intensive care unit; MELD, Model for End-Stage Liver Disease; D-MELD, the product of donor age and MELD; SOFT, Survival Outcome Following Liver Transplantation; BAR, Balance of Risk; TRI, Transplant Risk Index.

*Only 150 cases have the information of relationship

### Perioperative indexes

The length of hospitalization, length of intensive care unit (ICU) stay and time of mechanical ventilation were 50.2ength of hospitalization, length of intensive c (Table 1). The relationship between these indexes and different scores was analyzed (Table 2). The D-MELD score and BAR score displayed positive correlations with the length of ICU stay (D-MELD, p = 0.025; BAR, p = 0.022). Besides, there was a positive correlation between the SOFT score and the time of mechanical ventilation (p = 0.022).

**Table 2:** The correlation between the scores and perioperative indexes.

Scores	Length of hospitalization		Length of ICU stay		Time of mechanical ventilation	
	*r*	*p*	*r*	*p*	*r*	*p*
MELD	0.109	0.086	0.12	0.059	0.075	0.24
D-MELD	0.04	0.534	0.142	0.025	0.096	0.132
SOFT	-0.027	0.667	0.115	0.069	0.145	0.022
BAR	0.082	0.198	0.145	0.022	0.086	0.175
TRI	-0.095	0.135	0.123	0.052	0.08	0.207

Abbreviations: r, Spearman correlation coefficient; ICU, intensive care unit; MELD, Model for End-Stage Liver Disease; D-MELD, the product of donor age and MELD; SOFT, Survival Outcome Following Liver Transplantation; BAR, Balance of Risk; TRI, Transplant Risk Index.

### Postoperative complications

In this study, a total of 227 patients had the information of postoperative complications. Among them, 102 (44.9%) patients had early complications (≦30 days after LDLT) and 33 (14.5%) patients had late complications (>30 days after LDLT). The scores between patients with and without complications were compared and the results were shown in Table 3. By compared with patients without early complications, those with early complications had significantly higher D-MELD score, SOFT score and BAR score (p<0.05). However, no correlation was identified between late complications and all scoring systems.

**Table 3:** Postoperative Complications.

	D-MELD	SOFT	BAR	TRI
Early complications (≤30 days)				
YES (102)	646.3±467.6	2.19±3.9	4.8±4.7	6.4±12.5
NO (125)	511.7±340.0	0.8±3.4	3.3±3.7	4.7±2.8
*p*	0.013	<0.01	0.011	0.14
Late complications (>30 days)				
YES (33)	600.2±416.3	1.4±3.7	4.7±4.4	4.6±1.5
NO (194)	567.4±406.4	1.4±3.7	3.9±4.2	5.6±9.3
*p*	0.669	0.998	0.305	0.532

Abbreviations: D-MELD, the product of donor age and Model for End-Stage Liver Disease; SOFT, Survival Outcome Following Liver Transplantation; BAR, Balance of Risk; TRI, Transplant Risk Index.

### Pairwise comparison of the AUCs between the MELD and the other four scores

**Fig 1:**
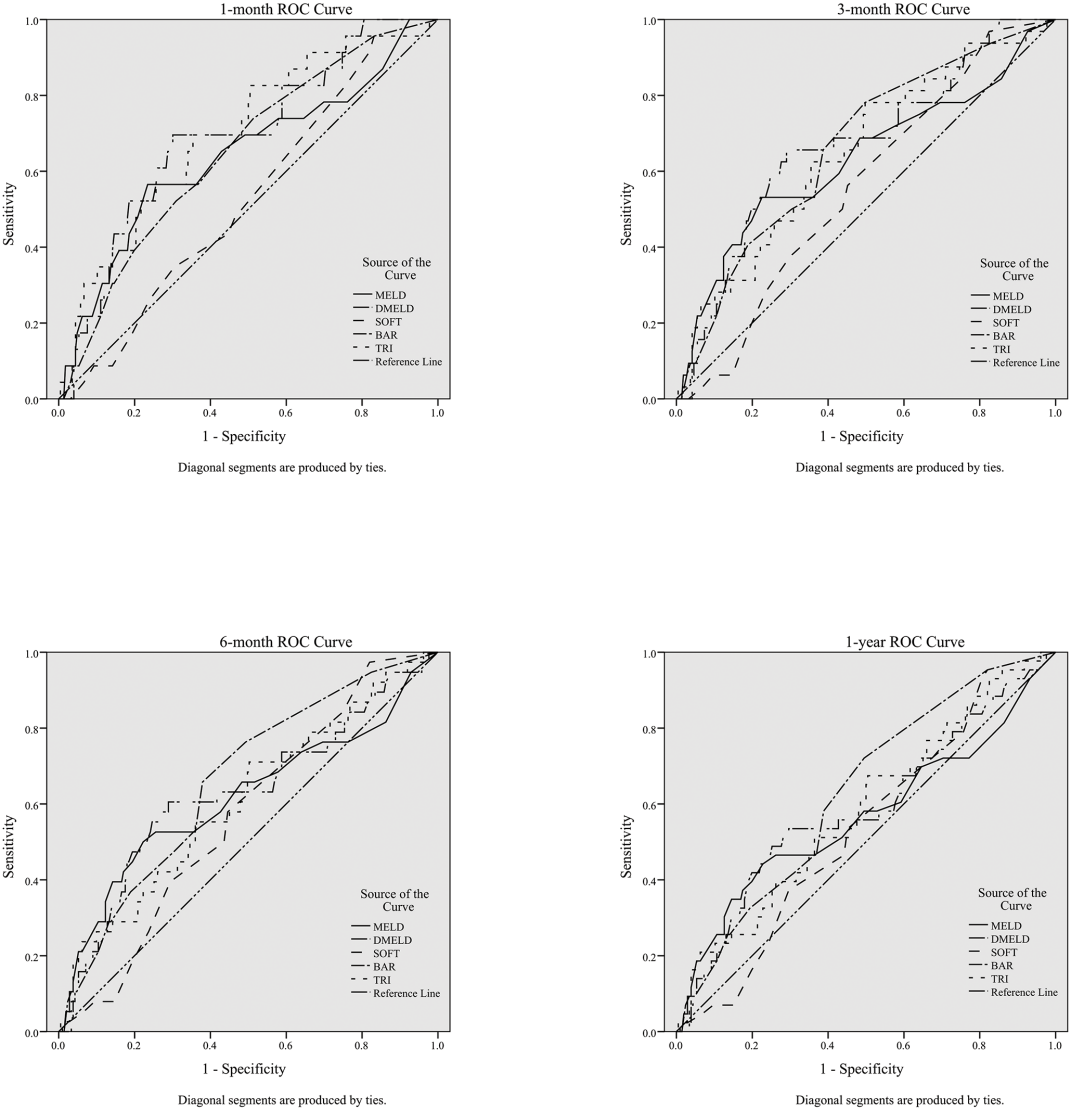
Comparison of ROC curves at 1-, 3-, 6-month and 1-year. Abbreviations: ROC, receiver operating characteristic curve; MELD, Model for End-Stage Liver Disease; D-MELD, the product of donor age and MELD; SOFT, Survival Outcome Following Liver Transplantation; BAR, Balance of Risk; TRI, Transplant Risk Index.

With the 1-, 3-, 6-month and 1-year as the endpoints, the ROCs of the five scoring systems are shown in Fig 1. The discrimination analysis showed the ability of different scores in predicting survival after LDLT (Table 4). The MELD score, D-MELD score and TRI score showed acceptable discriminative performances at 1-, 3- and 6-month after LDLT (AUCs>0.6), while the SOFT score showed poor discriminative performance at 6-month after LDLT. The performance of the BAR score was found to be steady, with all AUCs>0.6 at 1-, 3-, 6-month and 1-year after LDLT. Pairwise comparison revealed that the BAR score was more predictable than the MELD score at 1 year after LDLT (p = 0.046).

**Table 4:** Pairwise Comparison of ROC Curves between MELD and the Other Scores.

Scores	1 Month		3 Months		6 Months		1 Year	
	AUC (95% CI)	*P*	AUC (95% CI)	*P*	AUC (95% CI)	*P*	AUC (95% CI)	*P*
MELD	0.641 (0.579–0.701)	-	0.631 (0.568–0.691)	-	0.615 (0.551–0.675)	-	0.566 (0.502–0.629)	-
D-MELD	0.690 (0.629–0.747)	NS	0.669 (0.606–0.727)	NS	0.627 (0.564–0.687)	NS	0.588 (0.525–0.650)	NS
SOFT	0.528 (0.464–0.592)	NS	0.559 (0.495–0.622)	NS	0.569 (0.505–0.631)	NS	0.537 (0.473–0.600)	NS
BAR	0.651 (0.588–0.710)	NS	0.665 (0.602–0.723)	NS	0.660 (0.598–0.719)	NS	0.630 (0.567–0.691)	0.046
TRI	0.699 (0.637–0.755)	NS	0.650 (0.587–0.709)	NS	0.607 (0.543–0.668)	NS	0.584 (0.520–0.646)	NS

P values were calculated by comparing AUCs between the MELD and other scores.

Abbreviations: AUC, area under the receiver operating characteristic curve; CI, confidence interval; NS, non-significant; MELD, Model for End-Stage Liver Disease; D-MELD, the product of donor age and MELD; SOFT, Survival Outcome Following Liver Transplantation; BAR, Balance of Risk; TRI, Transplant Risk Index.

## Discussion

In countries where procurement from brain dead patient is prohibited by law, and in countries where the cadaveric organ donation rate is low, the LDLT is a good choice for patients with end-stage liver diseases. Usually, a living donor is uniquely matched to a certain recipient. Because of the special relationship between donors and recipients, even a patient in suboptimal conditions have the chance to undergo LDLT, such as a patient with advanced carcinoma [12]. Concerning risk to the living donor, LDLT should be performed more cautiously. To be responsible for both donors and recipients, a scientific score to predict outcome after LDLT is needed.

D-MELD, adding donor age into the MELD score, was developed in 2009 based on an analysis of 17 942 patients [3]. In our analysis, D-MELD displayed positive correlations with the length of ICU stay (p = 0.025) and the incidence of early postoperative complications (p = 0.013). Halldorson et al. reported that increasing D-MELD was strongly associated with progressively decreasing probability of survival. However, we have to point out that the authors performed only univariate analyses, without including several other factors which would affect posttransplant outcomes, such as recipient age, retransplant status, prior operation, or ischemia time [3]. A recent study by Ikegami et al. also showed that a D-MELD score of 462 had the highest sensitivity for predicting in-hospital mortality [13]. In addition, among a cohort of 303 consecutive adults, logistic regression did not show a significant correlation between graft failure and D-MELD score in the absence of a significant D-MELD cutoff [14]. Similar to this analysis, D-MELD score failed to predict short-term patient and graft survival by using a single-center 3-year German database [15]. It might be due to differences in candidates and donor pool, as compared to the original study.

In 2008, the SOFT score was developed and proved to accurately predict recipient posttransplant survival at 3 months with a C statistic of 0.70 [2]. However, inclusion of numerous covariates makes the score system less practicable. It could not be ignored that some variables, such as life supporter intensive care unit stay prior to transplantation, were overlapped with these factors as, for example, dialysis before transplantation, encephalopathy, ascites, and the need for ventilation [4]. Although the SOFT score was validated among cohorts of the sickest transplant candidates and the poorest-quality allografts by Rana et al., a study from Germany reported that the SOFT score failed to predict3-month and 1-year patient and graft survival in high risk liver transplant recipients with a MELD-score ≥ 30 [16,17]. In our analysis, even though the SOFT score displayed a positive correlation with the time of mechanical ventilation (p = 0.022), the SOFT score showed the poorest performance in predicting the survival after LDLT among the four scores. It might be due to the fact that the SOFT score was developed based on deceased donor liver transplantation (DDLT), and the factors of donor’s death and national allocation were not used for calculation in our study, which probably had influence on the result.

The BAR score incorporates six components, including donor age, recipient age, MELD score, retransplant status, cold ischemia time, and need of life support prior to transplant [4]. On the basis of the original article, the score ranges from 0 to 27, and the authors found a threshold of BAR 18 to best discriminate overall mortality at 5 years after liver transplantation. The score proved to be highly discriminatory in both the UNOS database and European Liver Transplant Registry (ELTR) database, was demonstrated superiority to other scores, such as the MELD score, D-MELD score, and SOFT score [4,18]. Jochmans et al. validated the BAR score with patients in Belgium between 2000 and 2010, and got the result that this score indeed provided a more accurate prediction of 1-and 5-year recipient survival after liver transplantation than the MELD score [19]. Similarly, our result suggested that BAR showed all AUCs>0.6 at 1-, 3-, 6-month and 1-year after LDLT. Besides, we found that the BAR displayed a positive correlation with the length of ICU stay (p = 0.022), and higher BAR score was associated with more early postoperative complications (p = 0.011).

The TRI score was proposed by Stey at al. in 2013, and in their study the predictive ability of TRI was proved to be greater than D-MELD [5]. According to the authors, the TRI captured recipient and donor factors not captured in UNOS, which improved the ability of good donor-recipient pairing. Our result suggested that the TRI was the most effective score in predicting 1-month mortality after LDLT among these four scores.

In 2001, Wiesner et al. had reported that the MELD score was widely used in liver transplantation for patient selection and organs allocation [20]. Today, the MELD score is still the major method to stratify liver transplantation recipients into different risk groups in China. Although the ability of MELD for predicting outcomes after LDLT is controversial [21–24], we found that the MELD score showed an acceptable discriminative performances in predicting survival at 1-, 3- and 6-month after LDLT. And our result also suggested that there is no significant difference between MELD and other four scores in predicting outcomes after LDLT. The reason why we failed to find differences between MELD and the other scores may be explained by the fact that all the donors used in the present study were health individuals, instead of deceased donors. In DDLT, the quality of grafts from different donors can vary significantly, which may contribute to different outcomes after LT. However, in LDLT, all the donors are healthy individual and the physical differences between individuals are small.

Besides, this study has two potential limitations. Firstly, this was a single-center and retrospective study. Secondly, all of the scoring systems above were developed from non-Chinese patients. Different from western countries, most Chinese patients were HBsAg-positive. Due to different clinical characteristics in different regions, the predictive ability of these scores may vary significantly.

In conclusion, the D-MELD, BAR and TRI scoring systems all provided acceptable prognostic ability to predict survival after LDLT. Although these scores included both donor and recipient characteristics, only the BAR score proved to provide a more accurate prediction of 1-year recipient survival after LDLT than the MELD score. However, further multicenter studies are anticipated to assess these results.
